# Characterization and Influence of Static In Vitro Digestion on Bioaccessibility of Bioactive Polyphenols from an Olive Leaf Extract

**DOI:** 10.3390/foods11050743

**Published:** 2022-03-03

**Authors:** Carmen Duque-Soto, Rosa Quirantes-Piné, Isabel Borrás-Linares, Antonio Segura-Carretero, Jesús Lozano-Sánchez

**Affiliations:** 1 Research and Development Functional Food Centre (CIDAF), Health Science Technological Park, Avenida del Conocimiento 37, Edificio BioRegión, 18016 Granada, Spain; carmenduque@correo.ugr.es (C.D.-S.); rquirantes@cidaf.es (R.Q.-P.); jesusls@ugr.es (J.L.-S.); 2 Department of Food Science and Nutrition, University of Granada, Campus Universitario s/n, 18071 Granada, Spain; 3 Department of Analytical Chemistry, Faculty of Sciences, Avda Fuentenueva s/n, University of Granada, 18071 Granada, Spain; ansegura@ugr.es

**Keywords:** olive leaf extract, polyphenols, HPLC, in vitro digestion, bioaccessibility

## Abstract

Olive leaves, one of the most abundant olive production by-products, have shown incredible potential for their characteristic bioactive compound composition, with unique compounds such as the polyphenol oleuropein. In order to evaluate the bioaccessibility of bioactive compounds present in an olive leaf extract, samples were submitted to an in vitro digestion process following INFOGEST protocol, and qualitative and quantitative characterization of the original extract and digestive samples at different times were carried out using HPLC-ESI-TOF-MS. The analyzed extract presented an abundance of phenolic compounds, such as secoiridoids, with oleuropein being the main identified compound. The in vitro digestion process showed an effect on the phenolic profile of the extract, with a lower recovery in the gastric phase and an increase at the beginning of the intestinal phase. Most of the studied compounds showed high bioaccessibility at the end of the digestion, with oleuropein, ligstroside, and quercetin-3-*O*-galactoside being among the ones with higher value. These findings show the potential for future use of olive leaf polyphenols. However, further research is needed in order to evaluate the absorption, delivery, and interaction of these compounds with the colon.

## 1. Introduction

The olive tree (*Olea europaea* L.) has been cultivated for centuries in the Mediterranean for the production of some of its most renowned products: oil and table olives. This area holds 98% of the crop area and 97% of the global production, with Spain being the first producing country [1,2]. As a result, abundant quantities of associated residues are produced. Only in Spain, 1–5 t/ha of the pruning residue in the form of leaves and branches are generated [1,3]. Its elimination has traditionally consisted of the grinding and burning of these tree-by-products. However, the environmental implications of these processes have led to an interest in the search for new innovative processing alternatives. In this respect, their reutilization has been proposed, as a way of propelling the circular economy and the development of products with high added-value.

As some of the most abundant by-products, the use of olive leaves for industrial purposes has been considered an innovative alternative, as this matrix poses as an interesting source of chemical compounds of great industrial potential. These structures present a wide variety of compounds ranging from mannitol, widely used as a sweetener for sugar-free products, to lignocellulosic compounds. In particular, there is a rising interest in their bioactive composition, from which phenolic compounds are the most important constituents.

Phenolic compounds are an extensive and heterogeneous group of molecules deriving from plants’ secondary metabolism, which have gained great interest in the scientific community in recent years. These molecules have demonstrated varied biological activities such as the antioxidant, anti-inflammatory, and antihypertensive effects related to some of the health benefits associated with olive leaves [4]. In this sense, olive leaf extracts rich in phenolic compounds are being studied for the treatment of diverse infections and as astringent and antiseptic agents [4,5,6]. Even though they have been considered for their therapeutic applications, the mentioned extracts could also be of great relevance for the food industry. Some of their pondered uses are in the production of functional foods or even as additives for the improvement of products’ shelf life, due to their natural action as antioxidants and preservatives. In this sense, they are already being evaluated for improving the oxidative stability of dietary oils [7].

Phenolic content in the olive tree may vary between different tree structures. Previous studies have shown a significantly higher concentration of these compounds in leaves than in other parts of the tree, which makes their extraction from this matrix of great interest to industry [7,8]. Due to their bioactive composition and its potential use, obtaining phenolic rich extracts from olive leaves and its conversion into high added-value ingredients for the food industry would suppose the promotion of a sustainable processing method of these olive by-products, generating natural nutritional products with minimal residue production, thus improving, and potentiating the circular economy.

However, in order to efficiently implement these extracts for their potential functional use, a wider knowledge of their chemical composition, biological activity, and behavior under gastrointestinal conditions is needed. The most popular technique for studying the phenolic profile of olive leaves in literature has been High Pressure Liquid Chromatography (HPLC) mainly coupled to mass spectrometry (MS). The bioactive compound profile of olive leaves is quite diverse, with oleuropein, a secoiridoid exclusive from the Oleaceae family, which represents from 60 to 90 mg/g of dry weight of leaves, being one of the most abundant [5,8]. Even though the isolation of single compounds could be considered, evidence on the synergistic effect of the interaction between polyphenols on its bioactivity shows a greater interest on the use of their corresponding extracts [6].

However, there still are challenges to its application in the food industry. As it is usually orally administrated, one of the most prominent challenges is the stability of the molecules of interest through the digestive tract, which influences its potential absorption and, therefore, hinders the accomplishment of their biological activity. In order to properly address this issue, the bioaccessibility and bioavailability of the compounds of interest must be considered, as those molecules need to reach their target areas before exerting their beneficial activity. Polyphenols are labile compounds, sensitive to both light and high temperatures, as well as rapidly metabolized and eliminated from the body. Thus, digestive conditions may contribute to a reduction in stability and directly affect their effectiveness, lowering their bioaccessibility and diminishing their absorption [7,9,10]. Additionally, absorption can also depend on their chemical structure, as some molecules might require previous metabolization in order to be accessible [4,7,11,12,13]. All these factors may affect and produce a different phenolic profile available for absorption than that observed in the olive leaf extract and should be studied in detail.

In order to study the bioaccessibility and bioavailability of phenolic compounds as influenced by the digestive process, in vivo approaches have been considered. Even though it seems to be a closer representation of the digestive process, there are disadvantages to their use, as a wide variety of parameters can alter the results, such as gender, age, or diverse disorders and physiological alterations. These models are also complex, expensive, and require extended periods of time [13,14]. These facts can also suppose an issue for the accurate and adequate interpretation of the bioaccessibility results, hindering the comparison between studies.

On the other hand, in vitro models have also been developed in an effort to reproduce the digestive process as close as possible. However, one of the main challenges is the variety of models developed to date. The variety and range of models and conditions considered hinder the accurate comparison between different studies. In order to solve these difficulties, the INFOGEST protocol was developed as the result of more than 2 years of discussion between multidisciplinary researchers to get a standardized and harmonized protocol of static in vitro digestion which allows comparison between studies. It applies constant ratios between food and digestive fluids at a constant pH for each digestive phase. Food samples are submitted to a sequential digestion consisting of oral, gastric, and intestinal phases, maintaining constants parameters such as electrolytes, concentration and enzymatic activity, bile, dilution, pH, and digestion time, based on physiological data and evidence [15].

Recently, a revision on this method has been published, called INFOGEST 2.0, which considers the possible problems associated with the original method, including an oral phase or the use of gastric lipase. This method also gathers some modifications for the study of the liberation of micronutrients during digestion. As for the evaluation of bioaccessibility of phytochemicals such as polyphenols, this model permits the realistic liberation of the compound to an aqueous phase [16]. However, in vitro digestion studies of polyphenol-rich olive leaf extracts seem to be scarce, and those available only monitor very few of these compounds. Additionally, as far as we are concerned, this study is one of the first to apply this method to olive-leaf extract.

Therefore, the aim of this study is the characterization of an olive leaf extract and the evaluation of the effect that the INFOGEST in vitro digestive protocol had on its polyphenolic profile, with the intention of identifying the resulting metabolites and the compounds available after the gastrointestinal process in order to determine their bioaccessibility.

## 2. Materials and Methods

### 2.1. Chemicals

All chemicals were of analytical reagent grade and used as received. Bovine bile salts (Sigma B-8631) and enzymes for in vitro digestion (pepsin 3412 U/mg protein and pancreatin 4xUSP) were obtained from Sigma Aldrich (Saint Louis, MO, USA). Sodium hydroxide (NaOH), hydrochloric acid (HCl), potassium chloride (KCl), potassium dihydrogen phosphate (KH_2_PO_4_), sodium hydrogen carbonate (NaHCO_3_), sodium chloride (NaCl), and ammonium carbonate ([(NH_4_)_2_CO_3_]) that were used to prepare the simulated digestive fluids were obtained from Fisher Chemicals (Waltham, MA, USA). HPLC–MS grade acetonitrile and formic acid were purchased from Fisher (Thermo Fisher Scientific, Leicestershire, UK). Standards of luteolin-7-*O*-glucoside (purity ≥ 98%), verbascoside (purity ≥ 99%), and loganin (purity ≥ 97.0%) were purchased from Sigma Aldrich, oleuropein (purity ≥ 98%) was purchased from Extrasynthese (Lyon, France), and hydroxytyrosol (purity ≥ 98%) was purchased from Cayman Chemical (Ann Arbor, MI, USA). Distilled water with a resistance of 18.2 MΩ was deionized in a Milli-Q system (Bedford, MA, USA).

### 2.2. Plant Material

Commercial olive leaf extract was provided by NATAC S.L., and it was obtained from the solid-liquid extraction of grinded leaves, with 80% ethanol as the extraction solvent with a solvent to sample ratio of 20:1, a temperature of 45 °C, and an extraction time of 2 h.

### 2.3. Static In Vitro Digestion INFOGEST

Static in vitro gastrointestinal digestion was performed following the INFOGEST 2.0 protocol described by Minekus et al. [15], taking into consideration the modifications provided by Brodkorb et al. [16] for the study of phenolic compounds, as well as the nature of the samples of study. The following process was carried out as a triplicate for each sample. In order to replicate oral digestion, 5 g of commercial olive-leaf dry extract were resuspended in 5 mL (1:1, *w*/*v*) of Simulated Salivary Fluid (SSF) in a 50 mL Falcon tube. This was stirred for 5 min, protecting the resultant mixture from light. For the gastric phase simulation, the bolus was mixed with 7.5 mL of Simulated Gastric Fluid (SGF), 2000 U/mL of pepsin, and 5 µL of CaCl_2_ 0.3 M. The pH was adjusted to 3.0, adding the necessary volumes of 1 M HCl. The final volume for this step was adjusted to 18 mL by addition of MilliQ H_2_O. The mixture was homogenized and inertized with N_2_. The gastric phase was carried out for 2 h at 37 °C under constant agitation at 150 rpm using a refrigerated incubator (MaxQTM 6000 SHKE6000-8CE, Thermo Scientific, Waltham, MA, USA). An aliquot of 1 mL was recovered and stored in an Eppendorf tube at the end of the present phase, labelled as GP.

For preparing the intestinal phase, 9.8 mL of Simulated Intestinal Fluid (SIF), 100 U/mL of pancreatin, 2.5 mL of bile, and 40 µL of CaCl_2_ 0.3 M were added to the existent simulated chyme. Then, the pH was fixed to 7.0, adding the required volumes of 1 M NaOH, and MilliQ H_2_O was added to achieve a final volume of 40 mL. This was homogenized and inertized with N_2_. The intestinal phase was carried out for 2 h at 37 °C under conditions of constant agitation at 150 rpm using a refrigerated incubator (MaxQTM 6000 SHKE6000-8CE, Thermo Scientific, Waltham, MA, USA). Aliquots of 1 mL were recovered at 30 min intervals and stored in an Eppendorf tube, labelled as IP1, IP2, IP3, and IP4.

For pH controlling purposes, through both the gastric and intestinal phases, pH measurements were at 30 min intervals, adjusting the value to 3.0 and 7.0, respectively, when necessary. Sample tubes were stored at −80 °C until further use. This process was carried out as a triplicate.

### 2.4. Bioaccessibility

Bioaccessibility, corresponding to the fraction of phenolic compounds freed from its food matrix into the gastrointestinal tract and, therefore, accessible for intestinal absorption, has been calculated using Equation (1) [17]. For each digestive phase, the accumulative presence of these compounds in the bioaccessible fraction was expressed as a percentage of the initial phenolic content, that is, according to the initial composition of the extract, using Equation (2) [18]. In this case, recovery at 240 min constitutes the final bioaccessibillity of each considered compound. In order to determine the initial phenolic content of the original extract to calculate the Bioaccessibility (Equation (1)), 5 g of extract was resuspended in the final volume of the intestinal phase (18 mL) and subjected to the sample treatment described for the bioaccessible fractions (Section 2.3):

(1) $$\mathrm{Bioaccessibility}\;\left(\%\right)=\frac{\mathrm{PC}\;\mathrm{content}\;\mathrm{in}\;\mathrm{IP}4\;\left(\mathrm{mg}\right)}{\mathrm{Initial}\;\mathrm{PC}\;\mathrm{content}\;\left(\mathrm{mg}\right)}\times 100\%\;$$

(2) $$\mathrm{Recovery}\;\left(\%\right)=\frac{\mathrm{PC}\;\mathrm{content}\;\mathrm{in}\;\mathrm{DS}\;\left(\mathrm{mg}\right)}{\mathrm{Initial}\;\mathrm{PC}\;\mathrm{content}\;\left(\mathrm{mg}\right)}\times 100\%\;$$

where PC is the phenolic compounds; IP4 is the final aliquot of the intestinal phase; DS is the digested samples; and Initial PC content refers to the presence of phenolic compounds in the olive leaf extract.

### 2.5. Bioactive Compound Extraction

Digested samples stored at −80 °C were processed before their characterization. For this purpose, samples stored in the Eppendorf tubes were defrosted in ice for 2 h and those in the Falcon tubes were stored overnight in the refrigerator. Samples GP, IP1, IP2, IP3, and IP4 (Gastric Phase and Intestinal Phase 1–4 taken at 150, 180, 210, and 240 min, respectively) were then homogenized and centrifuged at 14,800 rpm, 10 min, and 4 °C, conserving both bioaccessible (supernatants) and residual fractions (pellets).

For the extraction of phenolic compounds from the bioaccessible fraction, 200 μL were added to 100 μL of MeOH:EtOh 50:50 (*v/v*), agitated in vortex, and maintained at −20 °C for 30 min in order to precipitate the proteins. Then, the samples were centrifuged at 14,800 rpm, 10 min, and 4 °C, and the supernatants were evaporated in a vacuum concentrator for 4–5 h, later stored at −20 °C. Before characterization, 100 μL MeOH was added, and the resultant was homogenized in a refrigerated ultrasound bath for 4 h. After that, the processed digested samples were centrifuged under the previous conditions and the supernatants were introduced in HPLC vials for their later analysis.

As for the residual fraction, 1 mL of MeOH was added to 100 mg of residue, then homogenized and introduced in a refrigerated ultrasound bath for 15 min. Then, it was introduced into an incubator with agitation at 4 °C and centrifuged at 14,800 rpm, 10 min, and 4 °C. The supernatants were then evaporated in a vacuum concentrator for 2–3 h and stored at −20 °C. Before analysis, the samples were resuspended in a MeOH volume in order to achieve a concentration of 500 μg/mL, with the aid of a refrigerated ultrasound bath. Then, the processed residual fractions were centrifuged under the aforementioned conditions and the supernatants, diluted when necessary, were introduced in HPLC vials for their later analysis.

### 2.6. Characterization of Phenolic Compounds

For the characterization of the samples, stock solutions of 1 mg/mL in methanol were prepared from the following commercial compounds: hydroxytyrosol, oleuropein, verbascoside, luteolin-7-*O*-glucoside, and loganin. These solutions were filtrated using regenerated cellulose filters of 0.45 μm pore diameter and stored at −20 °C in amber screw cap bottles. Calibration curves with concentrations 1, 10, 25, 50, 100, and 150 μg/mL (hydroxytyrosol and oleuropein) and 1, 5, 10, 20, 30, and 40 μg/mL (verbascoside, luteolin-7-*O*-glucoside, and loganin) were prepared and analyzed as a triplicate.

Analyses were made using an Agilent 1200 Liquid Chromatography system (Agilent Technologies, Palo Alto, CA, USA) equipped with a micro vacuum degasser, binary pump, autosampler, thermostated column compartment, and diode array detector. The HPLC column used was an Agilent Zorbax Eclipse Plus C18 (1.8 μm, 4.6 × 150 mm). The mobile phases consisted of water plus 0.5% acetic acid (A) and acetonitrile (B). The multistep linear gradient applied was the following: 0 min, 5% B; 2 min, 30% B; 25 min, 95% B; 30 min, 95% B; and 42 min, 5% B. Then, the initial conditions were maintained for 3 min. The flow was 0.5 mL/min, temperature was maintained fixed at 30 °C, and injection volume in the HPLC system was 5 μL.

The HPLC system was coupled to a microTOF-Q II mass spectrometer (Bruker Daltoniks, Bremen, Germany) equipped with an ESI interface (Agilent Technologies, Palo Alto, CA, USA) operating in negative ion mode, in a mass range of 50–1000 *m/z*. Nitrogen was used as nebulizing/ionizing and drying gas at conditions of 2 bar and 10 L/min, respectively. Drying temperature was set at 190 °C, capillary voltage of +4 kV, and End Plate Offset at −500 V. Other optimum values for parameters were output voltage, 120 V; Skimmer 1, 40 V; Hexapole 1, 23 V; Hexapole RF, 100 Vpp; skimmer 2, 22.5 V; Lens 1 transfer, 50 µs; and Lens 1 Pre-Pulse Storage, 3 µs.

In order to recalibrate the mass spectra obtained during analysis to achieve a mass precision of 5 ppm, 5 mM sodium formate was used as a calibration agent at the beginning of each analysis, with an *m*/*z* range of 50–1200 Da.

### 2.7. Data Processing

For the phenolic compounds’ characterization, ion mass data were processed in the software DataAnalysis 4.0 (Bruker Daltoniks, Bremen, Germany), creating a molecular formulae list of the analyzed substances with a tolerance error of 2 ppm. Identification was carried out by comparison with literature and personal databases of phenolic compounds present in olive leaf, allowing for the identification of most of the compounds.

As for the quantification of both the extract and the different digested samples, chromatograms were also processed in DataAnalysis 4.0, where areas under each peak were calculated. Analyses were carried out in triplicate for each sample. Later, an adequate standard was selected for each compound according to their structural similarity, and its concentration was calculated by the interpolation of peak area detected in the replicate analysis of the samples in the calibration curve of the selected surrogate standard. Phenolic content in mg was calculated for each sample replicate and the mean concentration as well as statistic deviation was obtained. The quantitative content as well as the selected commercial standard for each identified compound are summarized in Supplementary Table S2 for bioaccesssible fractions and in Supplementary Table S3 for residual fractions.

## 3. Results

### 3.1. Olive Leaf Extract Characterization

Previous to the evaluation of the influence that the gastrointestinal conditions had on its chemical profile, it is necessary to evaluate the characterization of the extract of study, identifying all previous phenolic compounds of interest.

**Figure 1:**
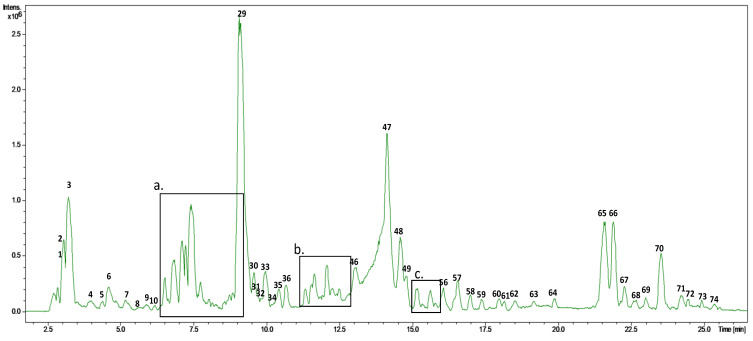

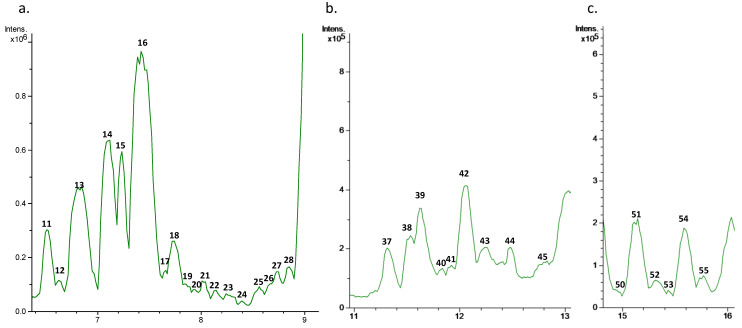
Base peak chromatogram (BPC, 50–1000 *m*/*z*) of the olive leaf extract, indicating zoomed zones (**a**–**c**).

Characterization of the olive leaf extract was carried out by HPLC-ESI-TOF-MS. As can be observed in Figure 1, the base peak chromatogram of this extract presented a high complexity. Therefore, major peaks were selected for their tentative identification, with a total of 74 compounds considered. Numbers were given according to retention time where, due to the nature of the chromatographic column, polar analytes were eluted at low retention times.

Identification was carried out by the comparison of retention times, *m*/*z* values, and molecular formulae proposed, and MS spectra provided by TOF-MS, with data provided by previous literature and personal databases of the phenolic compounds in olive leaf. Table 1 summarizes the MS data of the identified compounds, including the retention time, experimental and calculated *m*/*z* for the molecular formulae provided for each [M−H]^−^, and error, sigma value, and name of the proposed compound for each peak. MiliSigma value (mSigma) is a numeric value which indicates the level of similitude between theorical and experimental isotopic distributions, where a low value indicates statistical similitude. Its tolerance is normally established at 50, although factors such as coeluting analytes or compounds from the matrix could lead to higher values [19]. In this case, due to the complexity of the sample, some compounds achieved mSigma values higher than 50.

As can be observed in Table 1, most of the identified compounds belonged to the category of phenolic compounds, such as secoirioids, flavonoids, and simple phenols.

Secoiridoids. *Olea* *europaea* L. has presented an abundance of secoiridoids, such as oleosides, which are specific to this specie. In the extract of study, the most abundant compounds identified belonged to this class, as is the case for oleuropein (compound **29**), the most abundant and characteristic polyphenol found in this tree. This compound has been previously reported in literature as one of the main components of different tree structures, such as leaves, which is supported by this study, where it presented the most prominent peak by both intensity and area, which was later translated in a higher concentration [19,20,21,22,23,24,25,26,27]. Other isomers of oleuropein were also found at close retention times (compounds **27** and **30**). The extracted ion chromatogram (EIC) for *m*/*z* 701 presented a peak at retention time 7.85 (**19**), which has been identified as oleuropein diglucoside. Coinciding with the loss of two hexose residues (162 Da) in relation to the previous compound, 9 peaks were identified at *m*/*z* 377 and proposed as oleuropein aglycone in different isomeric forms (**34**, **36**, **39**, **43**, **45**–**49**). Additionally, two other oleuropein derivates were proposed such as hydroxyoleuropein (**22**), hydro-oleuropein (**26**), metoxyoleuropein (**28**), 10-hydroxyoleuropein aglycone (**40**), methyl oleuropein aglycone (**51**, **57**), and dimethyl oleuropein aglycone (**61**). Identification of these compounds has been confirmed with previous literature for the olive leaf [27,28], where 7 isomers of oleuropein aglycone were found in olive extracts.

Secoiridoids derivates from the structure of tyrosol have also been identified in this extract. A peak (**33**) with *m*/*z* 523 in EIC was found whose molecular formula corresponds with ligstroside, as well as an ion in EIC *m*/*z* 361 with ligstroside aglycone (**58**) [14,19,26,27,28,29,30]. Additionally, an [M-H]^−^ ion with *m*/*z* 389 (**14**) was assigned as oleoside or secologanoside, both of which have been previously identified in olive.

Simple phenols. Different simple phenols have been found in this extract, which have also been previously observed in different parts of the olive tree. Compound **15** has been identified as hydroxytyrosol, with a peak corresponding to an [M-H]^−^ ion with *m*/*z* 153. It has also been found in its glucoside form, corresponding with compound **13**, with an [M-H]^−^ ion with *m*/*z* 315 [20,21,22,25,27].

Other simple phenols were identified in the present extract as verbascoside/isoverbascoside (compound **18**, *m*/*z* 623) and hydroxyphenilacetic acid/vainillin (compound **7**, *m*/*z* 151) [14,19,29,31,32,33]. Peak 16 with *m*/*z* 377 (retention time 7.4 min) has been proposed as lamiol.

Flavonoids. Flavonoids are a characteristic group of compounds found in olive-tree related structures. Compounds from this class have been proposed as diosmetin-7-glucoside (**17**), three isomers of luteolin-glucoside (**20**, **21** and **25**), luteolin (**37**), quercetin-3-*O*-galactoside (**24**), and quercetin (**38**) [34].

Other compounds. There have been other compounds tentatively identified in the extract. Among them, we can find maslinic acid (**74**), widely described in olive leaf and related extracts, as well as compounds **10** and **12**, proposed as isomers of loganic/epiloganic acid, and compound **35**, identified as elenolic acid [35,36].

The [M-H]^−^ ion with an *m*/*z* 307 has been assigned as a phytoprostane, considered oxidative stress markers in superior plants [37]. Additionally, the peak found at EIC *m*/*z* 345 has been proposed as giberelic acid (**53**), a hormonal diterpene [38].

There have also been found oleanolic acid derivatives such as dihydroxy-oxo-oleanolic acid (**63**) and 11-oxooleanolic acid (**71**). The [M-H]^−^ ion with a 425 (**68**) has been proposed as 7-ketostigmasterol, a phytosterol, *m*/*z* 295 (**70**) as oxo-octadecenoic acid, and *m*/*z* 293 as diverse isomers of hydroxylindenic acid (**65**, **66**, **72**, **73**).

Carbohydrates and organic acids, such as sorbitol (**1**, **9**), glucuronic acid (**2**), citric acid (**4**, **6**), quinic acid (**3**), and rhamnosylarabinose (**8**, **11**), were proposed as compounds found at lower retention times. These are compounds found extensively in vegetal organisms, functioning as carbohydrate sources as well as metabolic intermediates, as citric acid is of the Krebs cycle. As well, at higher retention times, and therefore being more apolar compounds, different isomers of hydroperoxide 13-linolenic acid were found (**50**, **62**).

Unknown compounds. As shown in Table 1, the presence of compounds for which no structure has been able to be elucidated by the experimental evidence that was achieved in this study and bibliographic evidence can be observed (**5**, **23**, **31**, **32**, **41**, **42**, **52**, **55**, **56**, **59**, **60**, **64**, **67**, **69**). Some of these compounds have already been described for the studied vegetal matrix but have also been unsuccessful in their identification (**31**, **32**, **41**, **42**) [19,28].

### 3.2. Quantification of Phenolic Compounds in the Extract

Due to the great diversity of existing phenolic compounds, no commercial standards are available for all of them. Therefore, in order to quantify these compounds, a common approximation was applied using surrogate standard compounds with a similar enough chemical structure: oleuropein, hydroxytyrosol, luteolin-7-*O*-glucoside, verbascoside, and loganine. Thus, calibration curves for each of the surrogate standards were obtained using solutions described in Section 2.6 (Table S1). Chromatographic area of the detected peak for each compound was later substituted on the correspondent calibration curve of the selected standard based on structural similarity, obtaining the calculated concentration. Content in mg was calculated for each triplicate and the mean as well as statistic deviation was obtained. The quantification data as well as standards selected for each specific identified compound are present in Table S2 for bioaccessible fractions and Table S3 for residual fractions.

In Table 2, the concentration of these phenolic compounds can be observed, expressed as mean ± standard deviation for the three analyzed replicas.

The phenolic profile of the studied extract is consistent with previous literature for olive leaf extracts [28,29]. With 94% of the total quantified phenolic content, oleuropein and its isomers constitute the most abundant phenolic compounds found in the present olive leaf extract. Its concentration is superior to that of the rest of its derived compounds, which shows the predominance of free oleuropein as contrasted with literature [14,17,39]. The remaining phenolic compounds are found in a much lower concentration, as is the case for oleuropein aglycone (2%). It should also be noted that there was a higher concentration of hydroxytyrosol in its glycosylated form (1.1%) than in its free form (0.72%).

### 3.3. Influence of the In Vitro Digestive Process on the Phenolic Composition

In order to mimic the stability evolution of the bioactive compounds identified, a triplicate of the in vitro digestion of the olive leaf extract was carried out. During the digestive process, the samples were taken at the end of the gastric phase (GP) and for intervals of 30 min during the intestinal phase (IP), corresponding to times 120, 150, 180, 210, and 240 min, respectively.

The instability of polyphenols once extracted from its natural matrix make them highly vulnerable to the degradation mediated by surrounding conditions such as temperature and oxygen presence and food and gastrointestinal conditions (such as pH and enzymatic action), which can limit their absorption and, therefore, their bioaccessibility. These compounds are more stable at reduced temperature and pH, contrasting with the nature of the digestive conditions [40]. Therefore, gastrointestinal conditions could reduce their ability to reach a high enough concentration to be able to exert their health beneficial effect in their action zones [40,41].

Qualitative analysis showed that the phenolic profile seems to be constant through most of the in vitro gastrointestinal digestion process, being similar to the one found in the extract, maintaining most of the compounds through digested samples GP to IP4. However, that similarity is more noticeable in the gastric phase, where an isomer from both luteolin glucoside and oleuropein aglycone are missing from the initial profile. This difference is more pronounced in the intestinal digested samples, where a clear descent of oleuropein aglycone is observed, only maintaining isomer 2 by the end of the phase.

**Figure 2:**
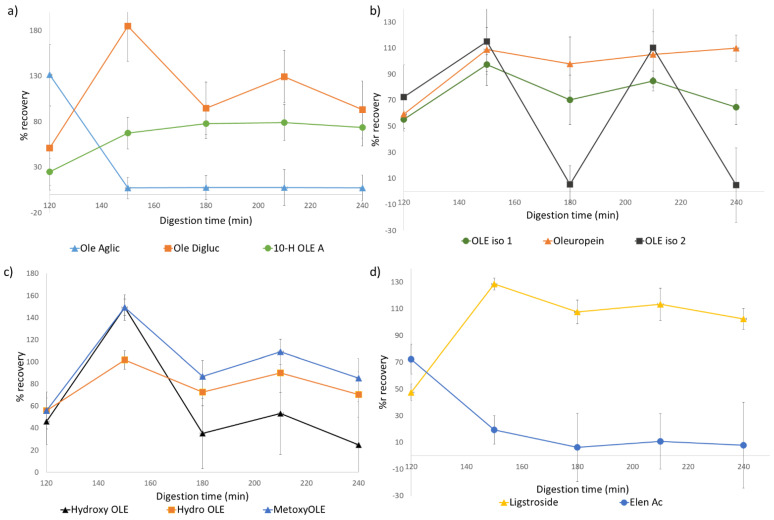

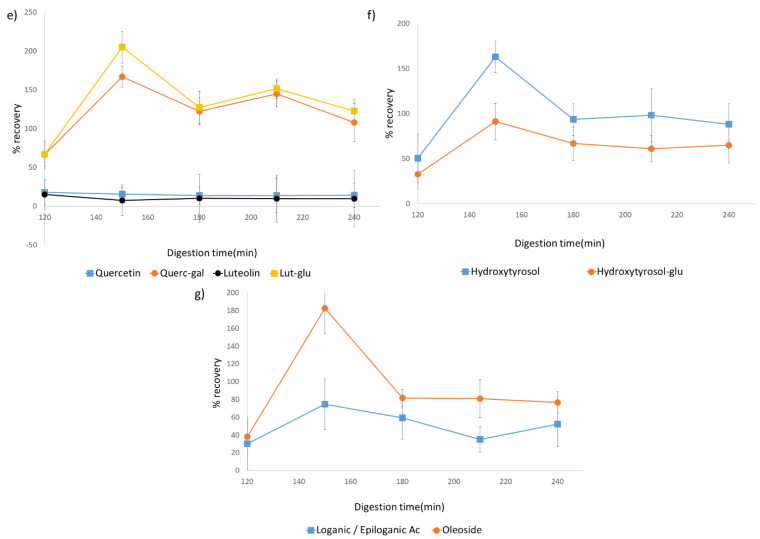
Accumulative recovery (%) of polyphenols during in vitro gastrointestinal digestion: (**a**) oleuropein aglycone, oleuropein diglucoside, and 10-hydroxyoleuropein aglycone; (**b**) hydroxyl-oleuropein and metoxyoleuropein; (**c**) oleuropein and isomers 1 and 2; (**d**) ligstroside and elenolic acid; (**e**) quercetin, quercetin-galactoside, luteolin, and luteolin-glucoside; (**f**) hydroxytyrosol and hydroxytyrosol glucoside; (**g**) loganic/epiloganic acid and oleoside.

The influence of the in vitro digestion can also be seen at a quantitative level. Differences have been found in the total amount of polyphenols quantified for different digestive phases and times. Additionally, differences were also found in comparison with the initial extract. Data from phenolic content of the bioaccessible fraction has been expressed as recovery percentage (Equation (2)), considering accumulative recovery of phenolic compounds from the resulting bioaccessible fractions of study, so that the final recovery rates at the end of the digestive simulation (240 min) correspond to the final bioaccessibility of each compound (Equation (1)). Detailed quantification data for the different compounds of each digested sample are present in the supporting information for bioaccessible (Supplementary Table S2I) and residual (Supplementary Table S3I) fractions. Kinetics of accumulative recovery during the in vitro digestion of olive leaf extracts are shown in Figure 2.

As for the total amount of phenolic content, we observed a tendency which was later seen in most of the monitored polyphenols. Expressed as recovery percentage, during the gastric phase, this value was significantly lower than in the rest of the simulation (60%). However, this differs from that observed for the intestinal samples. At the beginning of this phase (150 min), a dramatic increase in recovery was observed (185%), which was later reduced for subsequent sampling times. At 180, 210, and 240 min, these values were 75%, 124%, and 90%, respectively. Overall, the total quantified phenolic content was slightly reduced from the initial quantities, but this information gives no light into individual polyphenols behavior and variation throughout the simulation process.

In order to gain further insight into the process, influence on specific individual compounds was also considered. On the one hand, we found significant changes in oleuropein content in all digested samples. Initially, the presence of oleuropein and its different isomers was similar, increasing their recovery at the beginning of the intestinal phase in relation to the gastric phase, with values of 109% for oleuropein and 97% and 115% for both isomers. Through the intestinal phase, the presence of this compound lowered slightly at 180 min then increased, maintaining its concentration relatively stable. This profile was also observed for most of the phenolic compounds of study, such as ligstroside (Figure 2d), oleuropein derivatives (Figure 2a,c), hyroxytyrosol and its glycosylated form (Figure 2f), oleoside/secologanoside, and loganic/epiloganic acid (Figure 2g).

However, this behavior was much more instable for oleuropein isomer 2, where the decrease at 180 min was repeated at 240 min after an increase at 210 min. This behavior could be related to its close elution to oleuropein, which could lead to a partial overlap of both peaks, altering data reproducibility.

Additionally, some polyphenols showed a different behavior. An example is oleuropein aglycone which, as can be seen in Figure 2a, decreased drastically at the beginning of the intestinal phase and was kept stable through it. On the other hand, in the same graphic, the opposite behavior of 10-hydroxyoleuropein aglycone can be observed, with an increase in concentration during this phase. In a similar fashion, even though both hydroxytyrosol and its glycosylated form were found, showing a similar behavior as described, elenolic acid appeared to decrease significantly at the beginning of the intestinal phase, being relatively stable during the rest of the digestion process.

Evolution throughout the digestive process of quercetin and luteolin, as well as their galactoside and glucoside forms, respectively, can be observed in Figure 2c. While their galactoside and glucoside forms follow the previously described behavior, their free forms show a rather low recovery percentage, which was later stable during the rest of the process.

Finally, bioaccessibility for each of the selected compounds was calculated, as described in the Materials and Methods section. Bioaccessibility is the fraction of bioactive compounds, in this case phenolic compounds, which are freed from the administered formulation into the gastrointestinal tract and that, therefore, are available for their intestinal absorption [13].

For clarity purposes, the bioaccessibility of the selected phenolic compounds (corresponding to recovery at 240 min) is shown in Table 3. The digestive process shows a clear influence on the bioaccessibility of the phenolic compounds, as can be observed by the data present in Table 3. In general, a reduction on bioaccessibility was observed for the majority of the compounds. This is especially important for elenolic acid (7.9%), luteolin (9.9%), and quercetin (14.2%), which presented recovery percentages also low during the whole digestive process.

For some of the analyzed compounds, bioaccessibility was higher than 100%. This is the case of quercetin-3-*O*-galactoside (108%), oleuropein (109%), and ligstroside (102%). It must be noted that those values do not exceed greatly from 100%, and they could be related to the range of error associated with the sample taking and data processing, which could fit them inside a value slightly under. Nevertheless, this could also present a relation to variations on phenolic content throughout the digestion process. This could be related to the degradation of possible complexes of these molecules with other matrix compounds present in the extract that have not been identified during the characterization, as discussed in the next section. As bioaccessibility is calculated taking as a reference the initial content in mg of the initial extract, if during the digestive process non-detected complexes are degraded, the bioactivity percentage could be higher than 100%. Further discussion on this issue will be considered in the next section.

## 4. Discussion

Olive leaves, as abundant olive by-products, have been recently considered for their use as a natural source of bioactive compounds, such as phenolic compounds, which are of great interest for their health benefits. However, there is little research regarding their behavior under gastrointestinal conditions. Under in vitro static digestion, total phenolic content presented a variable tendency, with lower recovery during the gastric phase and an increase in the initial stages of the intestinal phase, which was later reduced. Additionally, most of the identified individual phenolic compounds showed a similar digestive profile as stated for the global behavior. Low recovery values at the gastric phase (120 min) could indicate the degradation of part of the bioactive compounds under gastric conditions due to the combination of pH and enzymatic effects. Nevertheless, the general behavior of the individual compounds showed an increase in its recovery at the beginning of the intestinal phase. This behavior has been previously described for some solid matrixes and olive leaf extracts [14,17,42,43]. This could be related to a low solubility of these compounds under gastric conditions. Experimental evidence obtained for residual fractions support this idea, as a high concentration of compounds in the gastric phase led to a drastic reduction of those in the intestinal phase.

Not only environmental conditions and solubility of compounds are decisive for its stability. The presence of compounds such as peptides and carbohydrates can also be a factor worth considering when evaluating the bioaccessibility of polyphenols. During gastrointestinal simulation, enzymes such as pepsin (gastric phase) and pancreatin (during intestinal phase), which is formed by α-amylase, pepsin, trypsin, and lipase, are introduced. As has already been observed in previous literature, phenolic compounds show an affinity to an amplitude of proteins and are able to form non-covalent complexes with proteins and, more specifically, enzymes such as those mentioned [44]. The influence of phenolic content on the activity of pepsin, presenting an inhibitory effect as a non-competitive inhibitor, show their capacity to establish specific interactions with this molecule [45,46]. It could be a possibility that phenolic compounds found in the extract interact closely with this enzyme during the gastric phase, which would not allow for these compounds to be detected or quantified under the selected HPLC conditions.

As these interactions have been shown to be influenced by an abundance of factors such as pH, concentration, type of phenolic compound, or temperature, changes in conditions from the gastric to intestinal phase, which consider alterations of pH and salinic concentration with the purpose of inhibiting a pepsin effect, could lead to the final separation of these PC-protein complexes. The possibility of the occurrence of these protein-phenolic compound complexes would also protect phenolic compounds from degradation, helping preserve the effective bioaccessibility and integrity of polyphenols, which could also coincide with the great bioaccesibility of these compounds.

When evaluating the bioaccessibility of polyphenols, the presence of peptides and carbohydrates (such as fibers) that could be in the extract even after extraction should also be taken into consideration. As seen in the characterization of the extract, some related molecules such as simple carbohydrates have been identified. It could be theorized that the presence, even if small, of higher molecular weight compounds interact with the present polyphenols that escape the applied analytical identification range. Such interactions have been previously described for flavonoids, which have the ability of interacting with fats, carbohydrates, or proteins of diverse nature [11]. However, these interactions have been mainly described in food matrices and, due to the scarcity of previous studies on the gastrointestinal simulation of olive leaf extract, this cannot be confirmed by other studies using similar matrices as the one considered in this study.

During the gastric and intestinal phases, different tendencies for the studied compounds can be observed, with a fluctuation of values associated with variations in pH conditions and possible presence of other compounds, as previously stated. In fact, in the present study, these alterations could be related with the presence of other components and their possible interactions with the bioactive compounds of interest. In some studies, great affinity of polyphenols with polysaccharides has been observed, such as those present in the cellular wall that may still be present in the extract as polysaccharides or fibers, such as pectins in the case of proanthocyanidins. This has deep consequences in the extractability of these compounds from their food matrices, and similar complexes in the correspondent extracts may be able to affect their bioaccessibility [47]. The disruption of cells during grinding and later extraction, puts in direct contact polyphenols with cell wall polysaccharides and polyphenol oxidase enzyme (PPO). This could lead to their oxidation catalyzed by the aforementioned enzyme, generating high molecular weight compounds which, although present, could not be detected under the mass range used. The incapacity for its detection and the degradation of compounds under gastric conditions could be responsible for the modulation in relative bioaccessibility [13].

This evidence could be supported by Ahmad-Qasem et al. (2014). In this study, a clear difference in behavior was observed between a standard solution of isolated polyphenols and olive leaf extracts under in vitro digestion conditions. An increase in antioxidant activity was observed in the extract after the gastric phase, related to a higher presence of polyphenols, reaching even higher values than before the digestion process. Oleuropein, verbascoside, and luteolin-7-*O*-glucoside standards submitted to the digestive process showed a decrease through digestion time. This can prove the importance that the interaction between polyphenols and compounds derived from their natural matrix has and their role in bioaccessibility.

Degradation of these complex structures during the intestinal phase can be explained through the high sensibility of dietary polyphenols to these conditions, with a higher pH than the gastric phase [48]. This would mean the degradation of complex molecules, probably associated with polysaccharides or proteins such as enzymes, allowing the increase of their free forms, in this case detectable and, therefore, justifying that high concentration. The sustaining of those conditions could also lead to a periodical degradation of free forms, as reflected in the evolution shown in the graphics.

As has been observed, some compounds present a different behavior. Oleuropein aglycone decrease and 10-hydroxyoleuropein aglycone increase could be related as a result of intestinal chemical reactions that benefit the hydroxylation of the first generating the second, which establishes the instability of oleuropein aglycone under intestinal conditions. The low recovery percentage of quercetin and luteolin could be related to the low solubility of flavonoids in aqueous media and their high stability.

Oleuropein being an ester of both elenolic acid and hydroxytyrosol is interesting in the evaluation of these degradation metabolites. In the digested samples, both hydroxytyrosol and its glycosylated form are found, showing a similar behavior as described. On the other hand, elenolic acid appears to decrease significantly at the beginning of the intestinal phase, being relatively stable during the rest of the digestion process. Oleuropein and derivatives reduction could be translated into an increase in both hydroxytyrosol and elenolic acid. However, the decrease of the latter could be indicative of instability of these compounds, which under intestinal conditions appears to suffer an intense degradation into other unknown or undetected molecules, modulating its bioaccessibility.

As for their bioaccessibility, due to the lack in literature regarding in vitro digestion of olive leaf extracts, these results have also been compared with those on other matrixes. Results from the previous study contrast with those presented by González et al. (2019) for an olive leaf extract, where bioaccessibility of oleuropein was lower than 50%, even though secoiridoids reached more than 300%. In López de Lacey et al. (2012), where bioaccessibility of quercetin-3-*O*-galactoside was evaluated in green tea extracts, the values were lower than ours, at 60%. For the rest of the phenolic compounds of study, this value is relatively high, i.e., above 50%, showing a great amount of them that can be absorbed, or at least be in the zone of absorption. Therefore, most of the phenolic content present in the extract can reach this area, being able to be absorbed into systemic circulation to later accomplish their biological activity. However, it is also known that most polyphenols can reach the colon, hence the mentioned compounds may be able to reach the mentioned area without being absorbed and interacting with colonic microbiota. For this reason, later studies focusing on the controlled delivery and release of polyphenols specifically to the colon, through the application of encapsulation techniques, would guarantee an increase in the active potential of these compounds.

On the other hand, the importance that the digestive process has on olive oil phenolic content has also been observed [49]. The observed bioaccessibility was rather low for secoiridoids, where hydrolysis of oleuropein derivatives led to values lower than 5%. In olive pomace, however, this parameter was closer to 100% for hydroxytyrosol, tyrosol, and oleuropein.

The nature of differences between these results could be due to variability in the plant source as well as extraction conditions. As some results consider polyphenols in oils, the difference in polarity could also alter the phenolic profile observed. In addition, the interaction with molecules such as fibers has been shown to alter bioaccessibility [50,51]. The diversity of in vitro digestion conditions applied for different studies must also be considered. This entails the introduction of multiple variability factors between studies that could influence stability and, therefore, the obtained results. Furthermore, the diversity in nature of the studied samples, as well as the differences in data expression, can also involve a higher effort into the comparison process.

The obtained data indicate the presence of significative concentrations of phenolic compounds in relation to those present in the extract that reach the intestinal phase and that, therefore, can be available for absorption. This constitutes a promising approach to the study of the impact of olive leaf polyphenols on human health. The influence of the intestinal absorption on its bioavailability, bioaccessibility, and bioactivity, also needs to be taken into consideration, as not all polyphenol structures are absorbed at the same rate. There are two main hypotheses on the intestinal absorption mechanisms: introduction through a Na+ dependent glucose transporters with a posterior deglycosylation and the absorption of aglycone forms by passive diffusion. Therefore, from the identified forms, only aglycones and glycosylated structures can be absorbed [52].

Although the in vitro static process gathers the most important conditions and phases of the digestion, the simplicity of itself makes it an adequate method of study for foods with simple compositions and purified components. Therefore, these data can constitute a preliminary study and sustain the continuation of research on bioaccessibility, taking into consideration a higher number of factors, such as the interaction with digestive microbiota. The presence and action of microbial enzymes could also present an impact on the final bioaccessibility of these molecules [13].

Part of the great importance of polyphenols on human health is derived from their action on microbiota, especially on its colonic phase. Non-absorbed phenolic compounds go into the large intestine, where they are degraded to phenolic acids by the action of the colonic microbiota [53]. This has raised an interest for its anti-inflammatory and immunomodulating activities in neoplasia, where the pro-inflammatory environment could promote carcinogenesis [54]. In this way, the non-absorbed fraction under intestinal conditions could still present an interest in the study of their potential health effects. Therefore, it can be considered as a future aspect for analysis of olive leaf extract polyphenols.

## 5. Conclusions

The phenolic profile of the present olive leaf extract was affected by the static in vitro digestion process. A total of 24 compounds have been monitored and their stability under gastrointestinal conditions has been studied. Degradation kinetics of major phenolic compounds in the bioaccessible fraction of samples showed similarity, with reduced concentration in the gastric phase, which increased in the first moments of the intestinal phase. Oleuropein and most derived compounds seem to present best recovery at the beginning of the intestinal phase, which was reduced at later times. This tendency has also been observed for the other monitored compounds. However, other compounds differ from this tendency, with quercetin and luteolin showing a low and stable recovery, as well as elenolic acid, which did not present a higher recovery at the beginning of the intestinal phase. During most of the intestinal phase, concentrations were stabilized, leading to high bioaccessibility values for most compounds such as oleuropein, the main compound identified, or ligstroside and quercetin-3-*O*-galactoside. Most of the studied compounds may be able to reach the colon, which may allow for future studies evaluating their stabilities under different colon segments, as well as their interaction with their correspondent microbiota.

## Figures and Tables

**Table 1:** Identified compounds in olive leaf extract.

Peak	RT (min)	Theoretical *m/z*	Formula	Measured *m/z*	Error (ppm)	mSigma	Proposed Compound
1	3.01	181.0732	C_6_H_13_O_6_	181.0718	−7.7	1.39	Sorbitol isomer 1
2	3.06	195.0528	C_6_H_11_O_7_	195.0510	−8.8	2.08	Glucuronic acid
3	3.30	191.0601	C_7_H_11_O_6_	191.0561	−20.7	25.67	Quinic acid
4	4.03	191.0224	C_6_H_7_O_7_	191.0197	−13.9	4.05	Citric acid isomer 1
5	4.40	421.1346	C_17_H_25_O_12_	421.1351	1.2	5.82	Unknown
6	4.63	191.0206	C_6_H_7_O_7_	191.0197	−4.8	2.02	Citric acid isomer 2
7	5.17	151.0400	C_8_H_7_O_3_	151.0401	0.3	18.49	Hydroxyphenylacetic acid/Vainillin
8	5.34	295.1029	C_11_H_19_O_9_	295.1035	1.9	24.55	Rhamnosylarabinose isomer 1
9	5.92	181.0718	C_6_H_13_O_6_	181.0718	−0.4	5.83	Sorbitol isomer 2
10	6.22	375.1289	C_16_H_23_O_10_	375.1297	2.0	14.10	Loganic/epiloganic acid isomer 1
11	6.57	295.1036	C_11_H_19_O_9_	295.1035	−0.4	4.76	Rhamnosylarabinose isomer 2
12	6.64	375.1294	C_16_H_23_O_10_	375.1297	0.6	23.03	Loganic/epiloganic acid isomer 2
13	6.84	315.1086	C_14_H_19_O_8_	315.1085	−0.1	8.24	Hydroxytyrosol glucoside
14	7.15	389.1114	C_16_H_21_O_11_	389.1089	−6.3	10.00	Oleoside/Secologanoside
15	7.25	153.0581	C_8_H_9_O_3_	153.0557	−15.8	15.20	Hydroxytyrosol
16	7.40	377.1451	C_16_H_25_O_10_	377.1453	0.5	15.65	Lamiol
17	7.67	461.1671	C_20_H_29_O_12_	461.1664	−1.3	18.50	Diosmetin−7-glucoside
18	7.82	623.2006	C_29_H_35_O_15_	623.1981	−3.9	1.27	Verbascoside/Isoverbascoside
19	7.87	701.2309	C_31_H_41_O_18_	701.2298	−1.6	8.61	Oleuropein diglucoside
20	7.97	447.0939	C_21_H_19_O_11_	447.0933	−1.4	15.50	Luteolin-glucoside isomer 1
21	8.05	447.0930	C_21_H_19_O_11_	447.0933	0.5	6.84	Luteolin-glucoside isomer 2
22	8.15	555.1730	C_18_H_35_O_14_	555.1778	−1.4	15.96	Hydroxyoleuropein
23	8.27	333.1552	C_15_H_25_O_8_	333.1555	0.7	12.65	Unknown
24	8.35	463.1475	C_19_H_27_O_13_	463.1457	−3.8	8.18	Quercetin-3-*O*-galactoside
25	8.55	447.0936	C_21_H_19_O_11_	447.0933	−0.8	11.33	Luteolin-glucoside isomer 3
26	8.67	541.1949	C_25_H_33_O_13_	541.1927	−4.2	8.90	Hydro-oleuropein
27	8.74	539.1784	C_25_H_31_O_13_	539.1770	−2.7	71.11	Oleuropein isomer 1
28	8.85	569.1879	C_26_H_33_O_14_	569.1876	−0.6	8.90	Metoxyoleuropein
29	9.07	539.1785	C_25_H_31_O_13_	539.1770	−1.6	24.76	Oleuropein
30	9.26	539.1779	C_25_H_31_O_13_	539.1770	1.3	4.37	Oleuropein isomer 2
31	9.56	601.2150	C_27_H_37_O_15_	601.2138	−2.1	16.07	Unknown
32	9.81	301.1293	C_14_H_21_O_7_	301.1293	−0.2	5.40	Unknown
33	9.99	523.1840	C_25_H_31_O_12_	523.1821	−3.6	13.64	Ligstroside
34	10.18	377.1233	C_19_H_21_O_8_	377.1242	2.2	24.41	Oleuropein aglycone isomer 1
35	10.41	241.0717	C_11_H_13_O_6_	241.0718	0.5	1.50	Elenolic acid
36	10.68	377.1252	C_19_H_21_O_8_	377.1242	−2.6	15.60	Oleuropein aglycone isomer 2
37	11.31	285.0395	C_15_H_9_O_6_	285.0405	3.2	42.95	Luteolin
38	11.55	301.0355	C_15_H_9_O_7_	301.0354	−0.6	2.56	Quercetin
39	11.66	377.1249	C_19_H_21_O_8_	377.1242	−1.9	14.63	Oleuropein aglycone isomer 3
40	11.85	393.1188	C_19_H_21_O_9_	393.1191	0.8	10.88	10-Hydroxyoleuropein aglycone
41	11.93	327.2167	C_18_H_31_O_5_	327.2177	3.0	28.79	Unknown
42	12.08	327.2175	C_18_H_31_O_5_	327.2177	0.5	4.41	Unknown
43	12.25	377.1241	C_19_H_21_O_8_	377.1242	0.2	18.41	Oleuropein aglycone isomer 4
44	12.48	331.2487	C_18_H_35_O_5_	331.2490	0.9	1.86	Trihydroxystearic acid
45	12.78	377.1237	C_19_H_21_O_8_	377.1242	1.4	18.55	Oleuropein aglycone isomer 5
46	13.04	377.1249	C_19_H_21_O_8_	377.1242	−1.9	14.33	Oleuropein aglycone isomer 6
47	14.11	377.1310	C_19_H_21_O_8_	377.1336	6.9	7.27	Oleuropein aglycone isomer 7
48	14.59	377.1261	C_19_H_21_O_8_	377.1242	−4.9	24.57	Oleuropein aglycone isomer 8
49	14.81	377.1248	C_19_H_21_O_8_	377.1242	−3.5	21.14	Oleuropein aglycone isomer 9
50	14.97	309.2045	C_18_H_29_O_4_	309.2071	8.4	22.65	13-hydroperoxide linolenic acid isomer 1
51	15.14	391.1380	C_20_H_23_O_8_	391.1398	4.8	8.53	Methyl oleuropein aglycone isomer 1
52	15.31	457.2785	C_31_H_37_O_3_	457.2748	−8.1	9.16	Unknown
53	15.46	345.1314	C_19_H_21_O_6_	345.1344	8.6	28.93	Gibberellic acid
54	15.63	307.1893	C_18_H_27_O_4_	307.1915	7.0	5.91	Phytoprostane
55	15.81	513.1740	C_27_H_29_O_10_	513.1766	5.1	7.91	Unknown
56	16.06	359.1123	C_26_H_15_O_2_	359.1078	−12.5	40.60	Unknown
57	16.55	391.1390	C_20_H_23_O_8_	391.1398	2.1	16.47	Methyl oleuropein aglycone isomer 2
58	16.95	361.1278	C_19_H_21_O_7_	361.1293	4.0	8.96	Ligstroside aglycone
59	17.38	305.1739	C_18_H_25_O_4_	305.1758	6.3	13.45	Unknown
60	17.95	721.3616	C_34_H_57_O_16_	721.3652	5.0	33.73	Unknown
61	18.15	405.1536	C_21_H_25_O_8_	405.1555	4.7	7.18	Dimethyl oleuropein aglycone
62	18.52	309.2057	C_18_H_29_O_4_	309.2071	4.8	7.78	13-Hydroperoxide linolenic acid isomer 2
63	19.14	485.3247	C_30_H_45_O_5_	485.3272	7.6	85.38	Dihydroxy-oxo-oleanolic/hydroxyoleanenedoic
64	19.86	647.3260	C_31_H_51_O_14_	647.3284	3.8	2.18	Unknown
65	21.53	293.2145	C_18_H_29_O_3_	293.2122	−7.9	47.09	Hydroxylindenic acid isomer 1
66	21.88	293.2149	C_18_H_29_O_3_	293.2122	−9.1	43.73	Hydroxylindenic acid isomer 2
67	22.28	487.2910	C_25_H_43_O_9_	487.2913	0.5	5.63	Unknown
68	22.55	425.3407	C_29_H_45_O_2_	425.3425	4.2	15.02	7-Ketostigmasterol
69	23.02	291.1954	C_18_H_27_O_3_	291.1966	4.0	35.63	Unknown
70	23.54	295.2281	C_18_H_31_O_3_	295.2279	−0.8	5.42	Oxo-octadecanoic acid
71	24.22	469.3307	C_30_H_45_O_4_	469.3323	3.4	57.77	11-Oxo-oleanolic acid
72	24.42	293.2115	C_18_H_29_O_3_	293.2122	2.6	15.79	Hydroxylindenic acid isomer 3
73	24.84	293.2108	C_18_H_29_O_3_	293.2122	4.8	1.13	Hydroxylindenic acid isomer 4
74	25.38	471.3456	C_30_H_47_O_4_	471.3480	5.0	16.94	Masilinic acid

**Table 2:** Quantification of selected compounds from olive leaf extract.

Phenolic Compounds	mg/g Dry Extract
Secoiridoids	
Oleuropein	76.1 ± 0.8
Oleuropein Isomer 1	0.094 ± 0.001
Oleuropein Isomer 2	13.0 ± 0.7
Oleuropein diglucoside	0.077 ± 0.01
Oleuropein aglycone Isomer 1	0.135 ± 0.001
Oleuropein aglycone Isomer 2	0.227 ± 0.008
Oleuropein aglycone Isomer 3	0.099 ± 0.008
Oleuropein aglycone Isomer 4	0.52 ± 0.06
Oleuropein aglycone Isomer 6	0.85 ± 0.08
Oleuropein aglycone Isomer 7	0.192 ± 0.007
Hydroxyoleuropein	0.035 ± 0.002
Hydro-oleuropein	0.065 ± 0.002
Metoxyoleuropein	0.084 ± 0.003
10-Hydroxyoleuropein aglycone	0.054 ± 0.002
Ligstroside	0.352 ± 0.008
Simple phenols	
Hydroxytyrosol	0.69 ± 0.07
Hydroxytyrosol glucoside	1.05 ± 0.07
Verbascoside/Isoverbascoside	0.246 ± 0.005
Hydroxyphenylacetic acid/Vainillin	0.11 ± 0.01
Flavonoids	
Luteolin	0.086 ± 0.006
Luteolin-glucoside Isomer 1 and 2	0.100 ± 0.003
Quercetin	0.142 ± 0.003
Quercetin-3-*O*-galactoside	0.019 ± 0.001
Diosmetin-7-glucoside	0.063 ± 0.002
Others	
Loganic/epiloganic acid Isomer 1	0.045± 0.002
Loganic/epiloganic acid Isomer 2	0.057 ± 0.004
Oleoside/Secologanoside	0.60 ± 0.01
Elenolic acid	0.165 ± 0.006
Total phenolic compounds	94 ± 2
Total polar compounds	95 ± 2

**Table 3:** Bioaccessibility percentage for the quantified phenolic compounds.

Phenolic Compounds	Bioaccessibility (%)
Secoiridoids	
Oleuropein	109.86
Isomer 1	64.68
Isomer 2	4.97
Oleuropein diglucoside	93.23
Oleuropein aglycone	64.64
Hydroxyoleuropein	24.63
Hydro-oleuropein	70.31
Metoxyoleuropein	85.36
10-Hydroxyoleuropein aglycone	73.68
Ligstrosid	102.29
Elenolic acid	7.95
Simple phenols	
Hydroxytyrosol	88.22
Hydroxytyrosol glucoside	65.06
Verbascoside/Isoverbascoside	72.93
Hydroxyphenylacetic acid/Vainillin	71.62
Flavonoids	
Luteolin	9.88
Luteolin-*O*-glucoside isomer 1 and isomer 2	92.90
Quercetin	14.21
Quercetin-3-*O*-galactoside	108.21
Diosmetin-7-glucoside	91.98
Others	
Loganic/epiloganic acid isomer 1	68.43
Loganic/epiloganic acid isomer 2	39.40
Oleoside/Secologanoside	76.72

## Data Availability

All the data generated by this research have been included in the article. For any assistance, it is possible to contact with the corresponding authors.

## Supplementary Materials

The following are available online at https://www.mdpi.com/article/10.3390/foods11050743/s1, Table S1: Main analytical parameters of calibration curves. Table S2: Identified compounds in the bioaccessible fraction expressed as total mg and standard deviation (SD) for each digestive phase. The used surrogate standard is indicated for each compound: Oleuropein (OLE), Luteolin-7-*O*-glucoside (LUT-GLU), Loganine (LOG), Hydroxytyrosol (HYTY), and Verbascoside (VERB). Table S3: Identified compounds in the residual fraction expressed as total mg and standard deviation (SD) for each digestive phase. The used surrogate standard is indicated for each compound: Oleuropein (OLE), Luteolin-7-*O*-glucoside (LUT-GLU), Loganine (LOG), Hydroxytyrosol (HYTY), and Verbascoside (VERB).
