# Supplementary material for: Characterization and Influence of Static In Vitro Digestion on Bioaccessibility of Bioactive Polyphenols from an Olive Leaf Extract
Source: Foods. 2022 Mar 3;11(5):743. doi: 10.3390/foods11050743 (PMC8909904; doi:10.3390/foods11050743)
Supplement: Supplementary file 1 [file foods-11-00743-s001.zip › foods-1611828-supplementary.pdf]

Table S1. Main analytical parameters of calibration curves.

Standard	Calibration range(μg/mL)	Calibration curve	R^2
**Oleuropein**	1-150	$y = -287.42x^2 + 95\,623.06x + 204\,183.66$	0.994
**Hydroxytirosol**	1-150	$y = -78.89x^2 + 52\,850.63x + 66\,152.16$	0.992
**Luteolin-7-O-glucoside**	1-40	$y = -914.83x^2 + 147\,880.00x + 137\,242.81$	0.990
**Verbascoside**	1-40	$y = 72\,057.68x + 26\,783.41$	0.996
**Loganin**	1-40	$y = -156.24x^2 + 133\,558.15x + 120\,127.47$	0.998

Table S2. Identified compounds in the bioaccesible fraction expressed as total mg and standard deviation (SD) for each digestive phase. The used surrogate standard is indicated for each compound: Oleuropein (OLE), Luteolin-7-O-glucoside (LUT-GLU), Loganine (LOG), Hydroxytyrosol (HYTY) and Verbascoside (VERB).

Compound	Standard	Extract		GP		IP1		IP2		IP3		IP4	
		mg	SD	mg	SD	mg	SD	mg	SD	mg	SD	mg	SD
**Hydroxyphenylacetic acid/Vainillin**	HY	0.58	0.06	0.32	0.04	0.6	0.1	0.41	0.07	0.5	0.1	0.42	0.08
**Loganic / epiloganic acid isomer 1**	LOG	0.23	0.01	0.08	0.02	0.19	0.04	0.18	0.03	0.095	0.009	0.16	0.03
**Hydroxytyrosol glucoside**	HY	5.3	0.3	1.8	0.2	4.9	0.7	3.6	0.4	3.3	0.3	3.5	0.5
**Loganic / epiloganic acid isomer 2**	LOG	0.29	0.02	0.07	0.02	0.20	0.05	0.13	0.03	0.084	0.008	0.11	0.02
**Oleoside/Secologanoside**	OLE	3.04	0.06	1.2	0.2	6	1	2.5	0.2	2.5	0.5	2.3	0.2
**Hydroxytyrosol**	HY	3.5	0.4	1.8	0.3	5.7	0.4	3.3	0.2	3.4	0.6	3.1	0.4
**Diosmetin-7-glucoside**	LUT-GLU	0.32	0.01	0.19	0.06	0.91	0.04	0.31	0.05	0.45	0.09	0.30	0.06
**Verbascoside/Isoverbascoside**	VERB	1.25	0.03	0.5	0.1	11	3	1.0	0.2	1.3	0.2	0.9	0.2
**Oleuropein diglucoside**	OLE	0.39	0.05	0.20	0.06	0.7	0.2	0.37	0.06	0.51	0.08	0.37	0.07
**Luteolin-7-O-glucoside isomer 2**	LU-GLU	0.29	0.01	0.10	0.01	0.41	0.08	0.27	0.04	0.32	0.03	0.27	0.03
**Hydroxyoleuropein**	OLE	0.178	0.009	0.08	0.01	0.27	0.02	0.06	0.02	0.10	0.03	0.04	0.02
**Quercetin-3-O-galactoside**	LUT-GLU	0.095	0.005	0.064	0.008	0.16	0.01	0.12	0.01	0.14	0.02	0.10	0.02
**Luteolin-7-O-glucoside isomer 3**	LUT-GLU	0.218	0.005	0.10	0.01	0.19	0.02	0.10	0.02	0.12	0.01	0.09	0.02
**Hydro-oleuropein**	OLE	0.33	0.01	0.18	0.03	0.34	0.02	0.24	0.02	0.30	0.04	0.23	0.04
**Oleuropein isomer 1**	OLE	0.476	0.006	0.26	0.01	0.46	0.03	0.34	0.06	0.40	0.03	0.31	0.04
**Metoxyoleuropein**	OLE	0.43	001	0.24	0.03	0.64	0.03	0.34	0.04	0.47	0.04	0.37	0.05
**Oleuropein**	OLE	387	4	230	30	421	67	380	70	410	70	430	40
**Oleuropein isomer 2**	OLE	65	4	48	9	76	20	3.5	0.3	70	20	3.3	0.7
**Oleuropein isomer 3**	OLE	-	-	0.31	0.03	0.47	0.07	0.44	0.08	0.48	0.05	0.43	0.04
**Ligstroside**	OLE	1.79	0.04	0.85	0.03	2.30	0.05	1.9	0.1	2.0	0.2	1.9	0.1
**Elenolic acid**	OLE	0.84	0.03	0.61	0.05	0.16	0.01	0.05	0.01	0.09	0.02	0.07	0.02

**Oleuropein aglycone isomer 1**	OLE	0.685	0.007	0.32	0.08	-	-	-	-	-	-	-	-
**Luteolin**	LUT-GLU	0.44	0.03	0.066	0.009	0.034	0.004	0.05	0.01	0.04	0.01	0.04	0.01
**Quercetin**	LUT-GLU	0.72	0.02	0.13	0.02	0.112	0.008	0.099	0.009	0.10	0.02	0.10	0.01
**Oleuropein aglycone isomer 2**	OLE	1.16	0.04	0.7	0.1	0.73	0.03	0.78	0.04	0.78	0.09	0.75	0.04
**10-Hydroxyoleuropein aglycone**	OLE	0.28	0.01	0.068	0.008	0.19	0.03	0.21	0.03	0.22	0.03	0.20	0.03
**Oleuropein aglycone isomer 3**	OLE	0.50	0.04	0.21	0.05	-	-	-	-	-	-	-	-
**Oleuropein aglycone isomer 4**	OLE	2.6	0.3	0.24	0.06	-	-	-	-	-	-	-	-
**Oleuropein aglycone isomer 5**	OLE	-	-	0.8	0.1	-	-	-	-	-	-	-	-
**Oleuropein aglycone isomer 6**	OLE	4.3	0.4	10	3	-	-	-	-	-	-	-	-
**Oleuropein aglycone isomer 7**	OLE	0.98	0.03	0.9	0.2	-	-	-	-	-	-	-	-
**Oleuropein aglycone isomer 8**	OLE	-	-	0.35	0.08	-	-	-	-	-	-	-	-

Table S3. Identified compounds in the residual fraction expressed as total mg and standard deviation (SD) for each digestive phase. The used surrogate standard is indicated for each compound: Oleuropein (OLE), Luteolin-7-O-glucoside (LUT-GLU), Loganine (LOG), Hydroxytyrosol (HYTY) and Verbascoside (VERB).

Compound	Standard	Extract		GP		IP1		IP2		IP3		IP4	
		Mean	SD	Mean	SD	Mean	SD	Mean	SD	Mean	SD	Mean	SD
**Hydroxyphenylacetic acid/Vainillin**	HY	0.58	0.06	0.4	0.2	0.28	0.04	0.21	0.06	0.21	0.04	0.09	0.02
**Loganic / epiloganic acid isomer 1**	LOG	0.23	0.01	0.17	0.02	0.109	0.009	0.11	0.01	0.106	0.007	0.069	0.008
**Loganic / epiloganic acid isomer 2**	LOG	0.29	0.02	0.2	0.1	0.099	0.008	0.10	0.01	0.091	0.008	0.067	0.007
**Hydroxytyrosol glucoside**	HY	5.3	0.4	1.9	0.3	4.8	0.6	4.2	0.3	4.1	0.4	3.4	0.2
**Oleoside/Secologanoside**	OLE	3.04	0.06	1.07	0.02	1.6	0.2	1.3	0.1	1.4	0.1	1.3	0.1
**Hydroxytyrosol**	HY	3.5	0.4	18	10	3.7	0.2	3.5	0.3	3.2	0.4	2.8	0.4
**Diosmetin-7-glucoside**	LUT-GLU	0.32	0.01	0.20	0.01	0.21	0.01	0.20	0.02	0.19	0.01	0.19	0.01
**Verbascoside/Isoverbascoside**	VERB	1.25	0.03	1.05	0.09	1.6	0.1	1.4	0.1	1.40	0.09	1.37	0.06
**Oleuropein diglucoside**	OLE	0.39	0.05	0.26	0.02	0.35	0.03	0.33	0.02	0.33	0.03	0.30	0.02
**Luteolin-7-O-glucoside isomer 2**	LUT-GLU	0.29	0.01	0.137	0.009	0.38	0.02	0.44	0.03	0.41	0.05	0.55	0.04
**Hydroxyoleuropein**	OLE	0.178	0.009	0.091	0.007	0.10	0.02	0.072	0.007	0.07	0.01	0.047	0.007
**Quercetin-3-O-galactoside**	LUT-GLU	0.095	0.005	0.034	0.003	0.11	0.03	0.08	0.02	0.074	0.008	0.045	0.008
**Luteolin-7-O-glucoside isomer 3**	LUT-GLU	0.218	0.005	0.06	0.03	0.211	0.008	0.221	0.008	0.19	0.02	0.218	0.009
**Hydro-oleuropein**	OLE	0.33	0.01	0.11	0.02	0.25	0.02	0.21	0.02	0.13	0.09	0.19	0.01
**Oleuropein**	OLE	0.476	0.006	0.19	0.03	0.37	0.04	0.31	0.03	0.2	0.2	0.27	0.02

**Metoxyoleuropein**	OLE	0.43	0.01	0.16	0.02	0.41	0.03	0.38	0.01	0.34	0.03	0.29	0.02
**Oleuropein isomer 1**	OLE	387	4	500	80	490	50	380	120	410	40	170	50
**Oleuropein isomer 2**	OLE	66	4	0.30	0.06	600	600	600	700	600	600	3.9	0.8
**Oleuropeína isómero 2**	OLE	-	-	-	-	0.7	0.1	0.64	0.08	2	2	0.57	0.04
**Ligstroside**	OLE	0.67	0.05	0.79	0.04	2.21	0.09	2.4	0.1	2.3	0.1	2.01	0.09
**Elenolic acid**	OLE	0.84	0.03	0.43	0.08	0.39	0.03	0.36	0.03	0.35	0.06	0.28	0.04
**Oleuropein aglycone isomer 2**	OLE	0.685	0.007	0.34	0.04	-	-	-	-	-	-	-	-
**Luteolin**	LUT-GLU	0.44	0.03	0.22	0.02	0.34	0.03	0.26	0.04	0.25	0.01	0.28	0.04
**Quercetin**	LUT-GLU	0.72	0.02	0.32	0.03	0.12	0.04	0.048	0.007	0.02	0.01	0.024	0.008
**Oleuropein aglycone isomer 3**	OLE	1.16	0.04	0.92	0.05	1.3	0.2	1.23	0.06	1.2	0.2	0.90	0.07
**10-Hydroxyoleuropein aglycone**	OLE	0.28	0.01	0.324	0.009	0.11	0.02	0.09	0.01	0.07	0.01	0.07	0.01
**Oleuropein aglycone isomer 4**	OLE	0.50	0.04	0.9	0.2	-	-	4.1	0.9	2.4	0.3	0.7	0.5
**Oleuropein aglycone isomer 5**	OLE	2.6	0.3	1.0	0.2	0.2	0.1	0.13	0.05	-	-	-	-
**Oleuropein aglycone isomer 6**	OLE	-	-	100	10	-	-	-	-	-	-	-	-
**Oleuropein aglycone isomer 7**	OLE	4.3	0.4	7	7	-	-	-	-	-	-	-	-
